# Supplementary material for: Alternative ergosterol biosynthetic pathways confer antifungal drug resistance in the human pathogens within the Mucor species complex
Source: mBio. 2024 Jul 9;15(8):e01661-24. doi: 10.1128/mbio.01661-24 (PMC11323496; doi:10.1128/mbio.01661-24)
Supplement: Supplemental Figures — Figures S1-S6. [file mbio.01661-24-s0001.pdf]

Figure S1

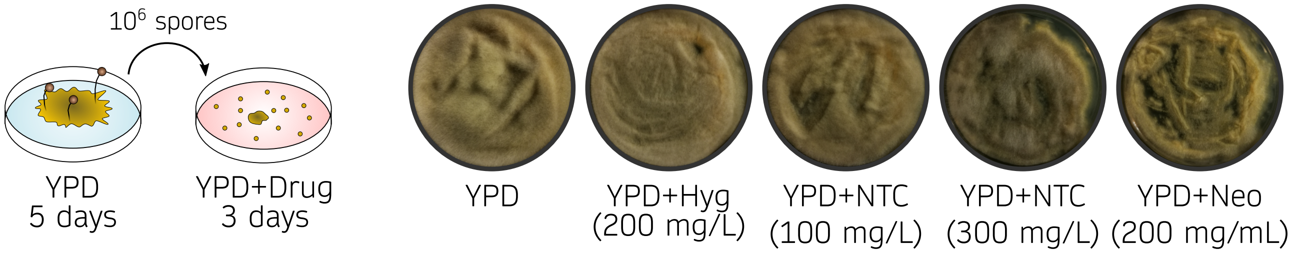

Figure S1. Evaluation of dominant resistance markers in *M. circinelloides*. 10^6 spores from *M. circinelloides* wildtype strain growing for 48 hours on YPD medium supplemented with Hygromycin (Hyg), Nourseothricin (NTC), and Neomycin (Neo) at the indicated concentrations.

Figure S2

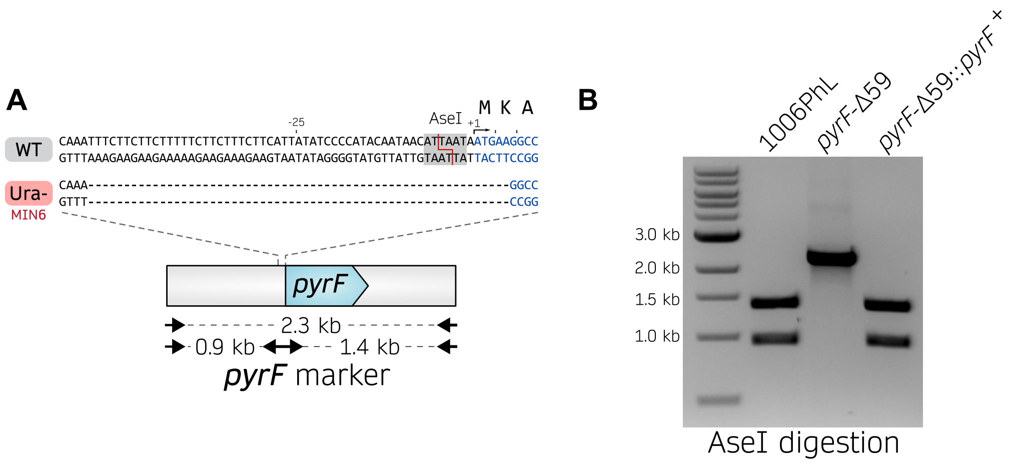

Figure S2. Complementation of the *pyrF* mutant allele in MIN6. (A) Wildtype and MIN6 mutant sequences for *pyrF*. The AseI restriction site is indicated in the wildtype 5' region of the *pyrF* gene, while this restriction site is missing in MIN6. **(B)** PCR-amplification followed by AseI digestion confirming *pyrF* complementation.

Figure S3

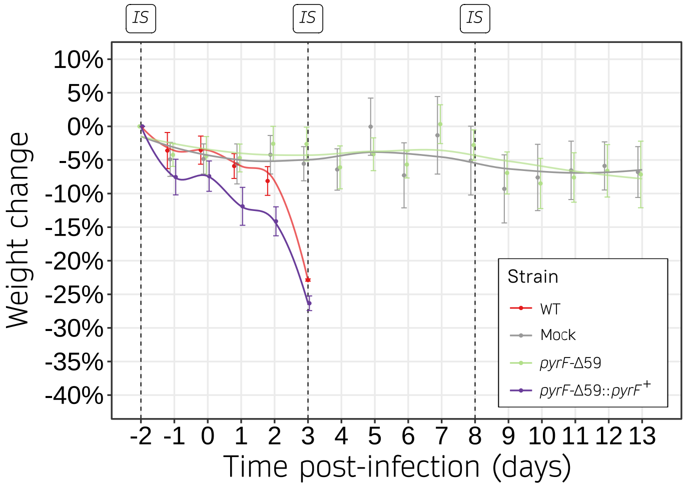

Figure S3. Weight monitoring of mice infected with uracil auxotrophic and prototrophic strains. Body weight of animals infected with wildtype 1006PhL, *pyrF*-59Δ auxotrophic strain, and *pyrF*-59Δ::*pyrF*$^+$ complemented strain. Animals infected with prototrophic strains (red and purple) show increased weight loss over time compared to animals infected with the auxotrophic strain (green) or PBS (gray). Immunosuppressive (IS) treatments are indicated as dotted lines.

Figure S4

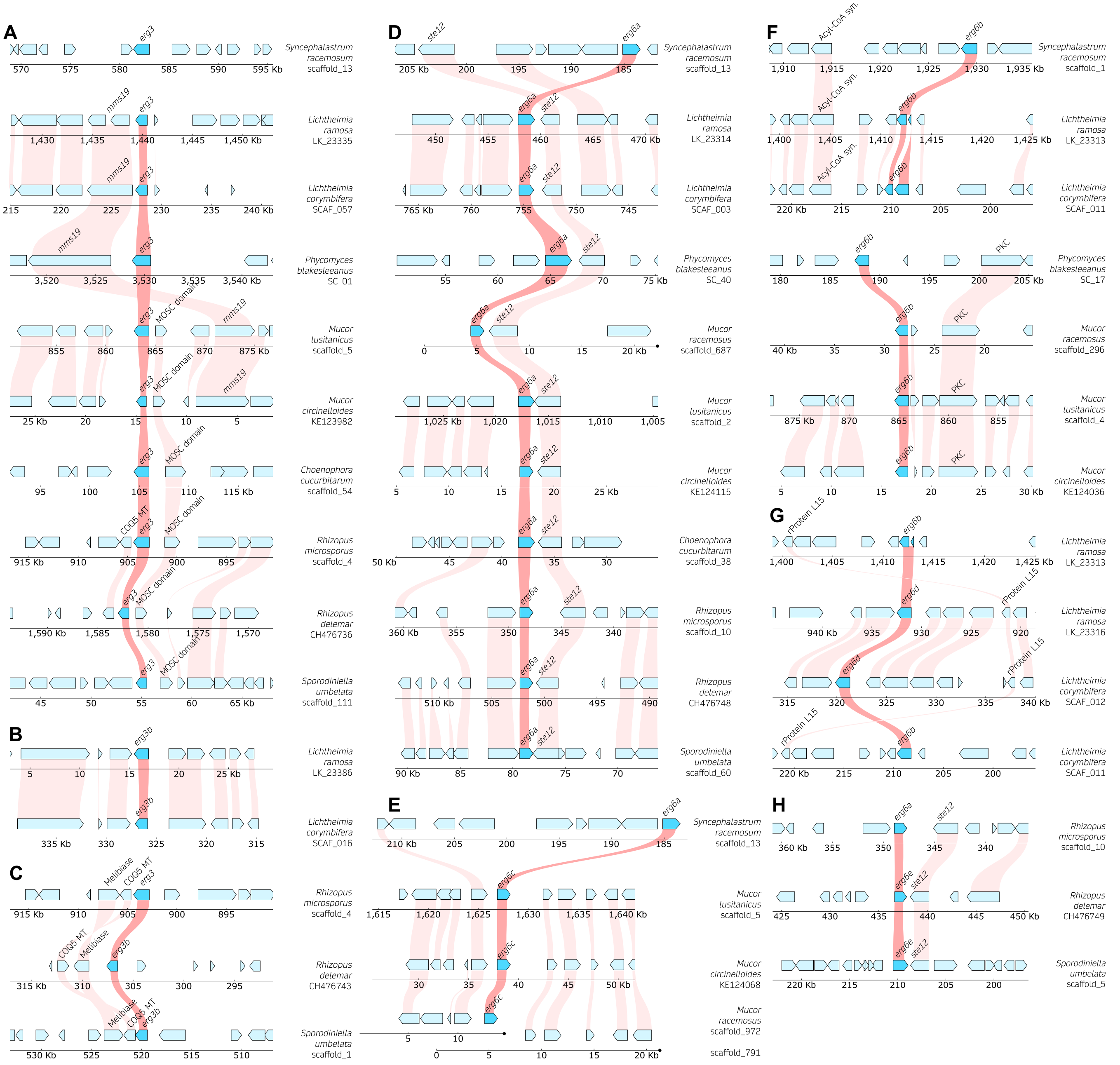

Figure S4. Synteny of *erg3* and *erg6* orthologs within the Mucoromycota. Gene synteny of the indicated species and chromosome locations of genes **(A)** *erg3*, **(B)** *erg3b* in *Lichtheimia* spp., **(C)** *erg3b* in *Rhizopus* spp., **(D)** *erg6a*, **(E)** *erg6c*, **(F)** *erg6b*, **(G)** *erg6d*, and **(H)** *erg6e*. Species pairwise comparisons were selected according to closest phylogenetic relationship and when possible, arranged similarly to species in clade *erg6a* from Figure 2B. The genome coordinates are indicated for each genome, and gene annotation is depicted as blue blocks highlighting the *erg* genes in cyan blue. Interspecies synteny among genes is depicted as red shading for *erg* genes and as pink shading for neighboring genes.

Figure S5

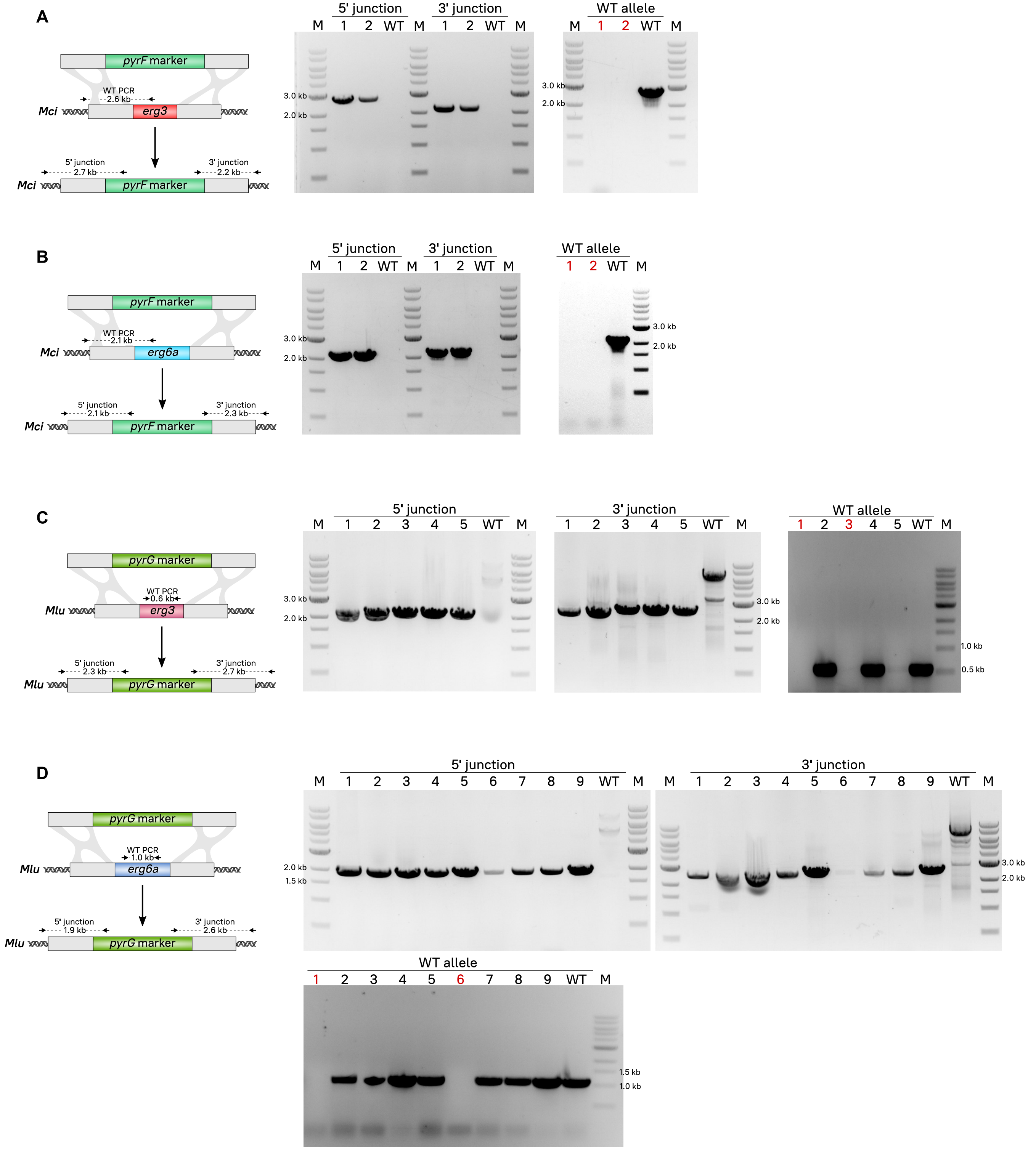

Figure S5. Generation of mutant strains for *erg3* and *erg6a* genes in *M. circinelloides* and *M. lusitanicus*. Diagram (left) and AFLP agarose gel images (right) showing the integration of either the *pyrF* or *pyrG* marker replacing the corresponding *erg* gene: deletion of *erg3* **(A)** and *erg6a* **(B)** in *M. circinelloides*; deletion of *erg3* **(C)** and *erg6a* **(D)** in *M. lusitanicus.* Lanes are numbered to indicate transformant isolates, except for M lanes that show a DNA ladder for fragment size comparison. Arrows mark the annealing regions for specific primers used to confirm the integration by two different PCR reactions: 5' junction, and 3' junction. Mutant homokaryosis was confirmed by absence of the wildtype (WT) allele, and homokaryotic mutants generated by independent transformation experiments are highlighted in red.

Figure S6

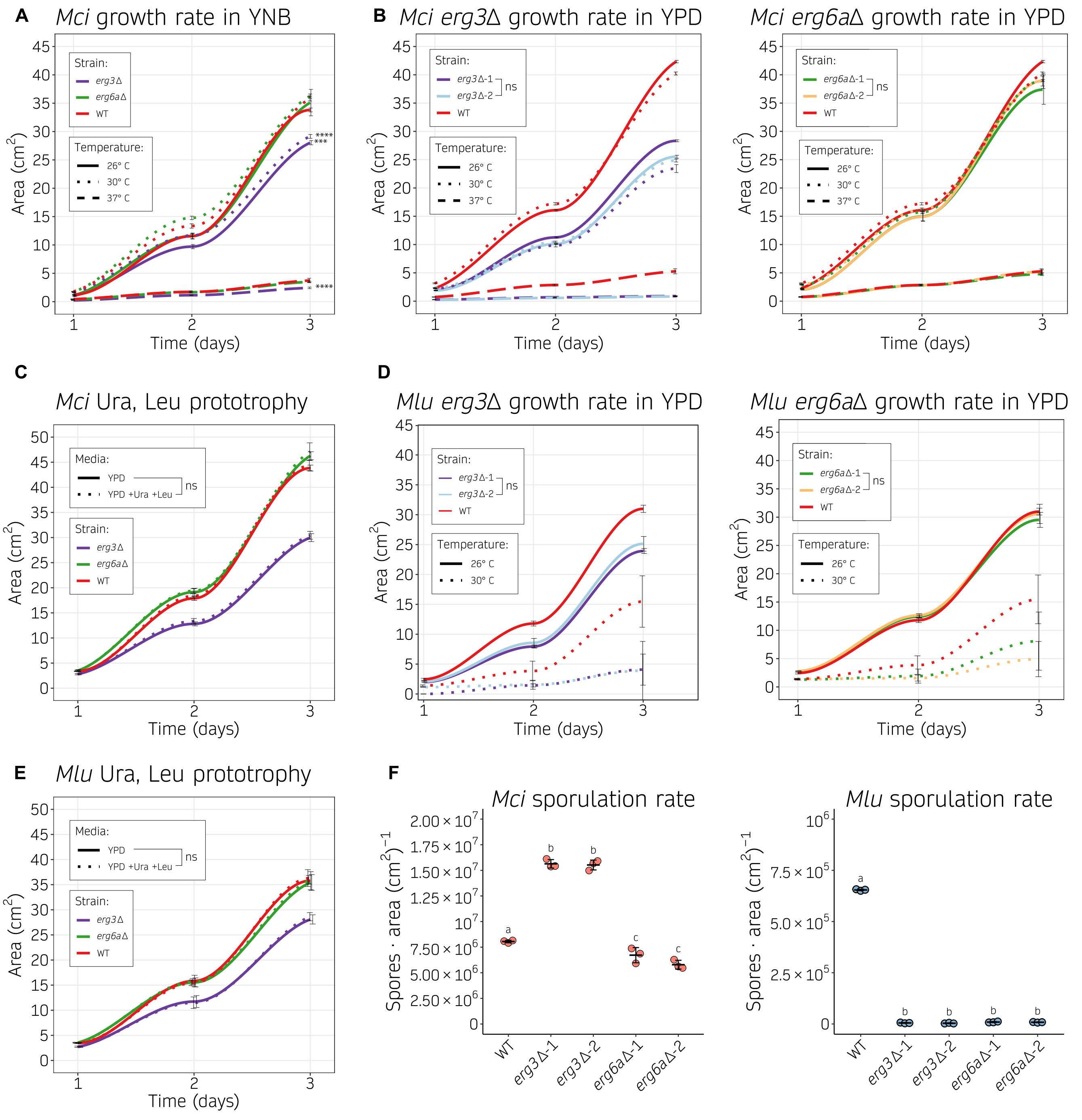

Figure S6. Growth and sporulation rates of *Mucor* species *erg3*Δ and *erg6a*Δ independently generated mutants. (A-E) Growth rate of *Mucor* species strains is depicted as color-coded lines, and temperature or media supplementation are indicated as distinct line types. The growth rate was determined as the area of growth after 24-hour periods. Each experiment was done using six replicates for the wildtype strain (WT), or three biological replicates for each pair of independently generated mutants (Δ-1 and Δ-2) accounting for six total replicates for each deletion and condition tested. Values are plotted as a smoothed curve and SD values as black lines. Growth rate was determined in mutant strains from **(A)** *M. circinelloides* growing in minimal medium YNB; **(B)** *M. circinelloides* in rich medium YPD; **(C)** *M. circinelloides* in rich medium YPD with or without uridine and leucine supplementation; **(D)** *M. lusitanicus* in rich medium YPD; and **(E)** *M. lusitanicus* in rich medium YPD with or without uridine and leucine supplementation. One-way ANOVA and Tukey HSD test were conducted for each temperature group and shown as asterisks (** *p-value* $\leq$ 0.01, *** *p-value* $\leq$ 0.001, and **** *p-value* $\leq$ 0.0001) in **(A)**, and to highlight that independently generated mutants harboring the same deletion did not show significant differences (ns) in **(B-D)**, as well as validating the strains prototrophy by showing no significant differences (ns) with uridine and leucine supplementation in **(C-E)**. **(F)** Sporulation rate from *M. circinelloides* or *M. lusitanicus* wildtype strains and two *erg3*Δ or *erg6a*Δ independently generated mutants (Δ-1 and Δ-2) was assessed as the number of spores per cm^2 after growing in MMC medium for 48 hours. Strains are grouped by letters, each group showing significant sporulation differences between them (One-way ANOVA and Tukey HSD test).
