# Supplementary material for: Alternative ergosterol biosynthetic pathways confer antifungal drug resistance in the human pathogens within the Mucor species complex
Source: mBio. 2024 Jul 9;15(8):e01661-24. doi: 10.1128/mbio.01661-24 (PMC11323496; doi:10.1128/mbio.01661-24)
Supplement: Supplemental Figures — Figures S7-S11. [file mbio.01661-24-s0002.pdf]

Figure S7

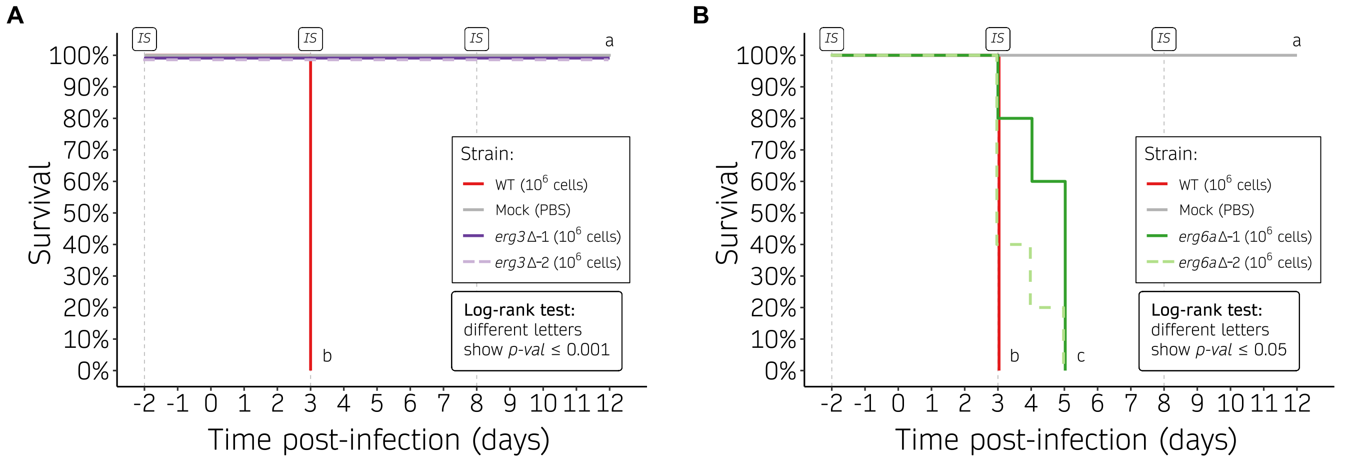

Figure S7. Survival curves of mice infected with *M. circinelloides erg3*Δ and *erg6a*Δ independently generated mutants. Kaplan-Meier survival curves of immunosuppressed mice infected with 10^6 spores from the wildtype strain and either **(A)** *erg3*Δ-1 and *erg3*Δ-2 mutants, or **(B)** *erg6a*Δ-1 and *erg6a*Δ-2. Groups of five mice were inoculated with each independently generated mutant, accounting for ten total mice per deletion or wildtype strain. Strains showing significant differences in virulence assessed by a Log-rank test are indicated as different letters (*p-value* $\leq$ 0.05), confirming that independently generated mutants harboring the same deletion did not show significant differences in virulence. Immunosuppressive treatments are indicated (IS).

Figure S8

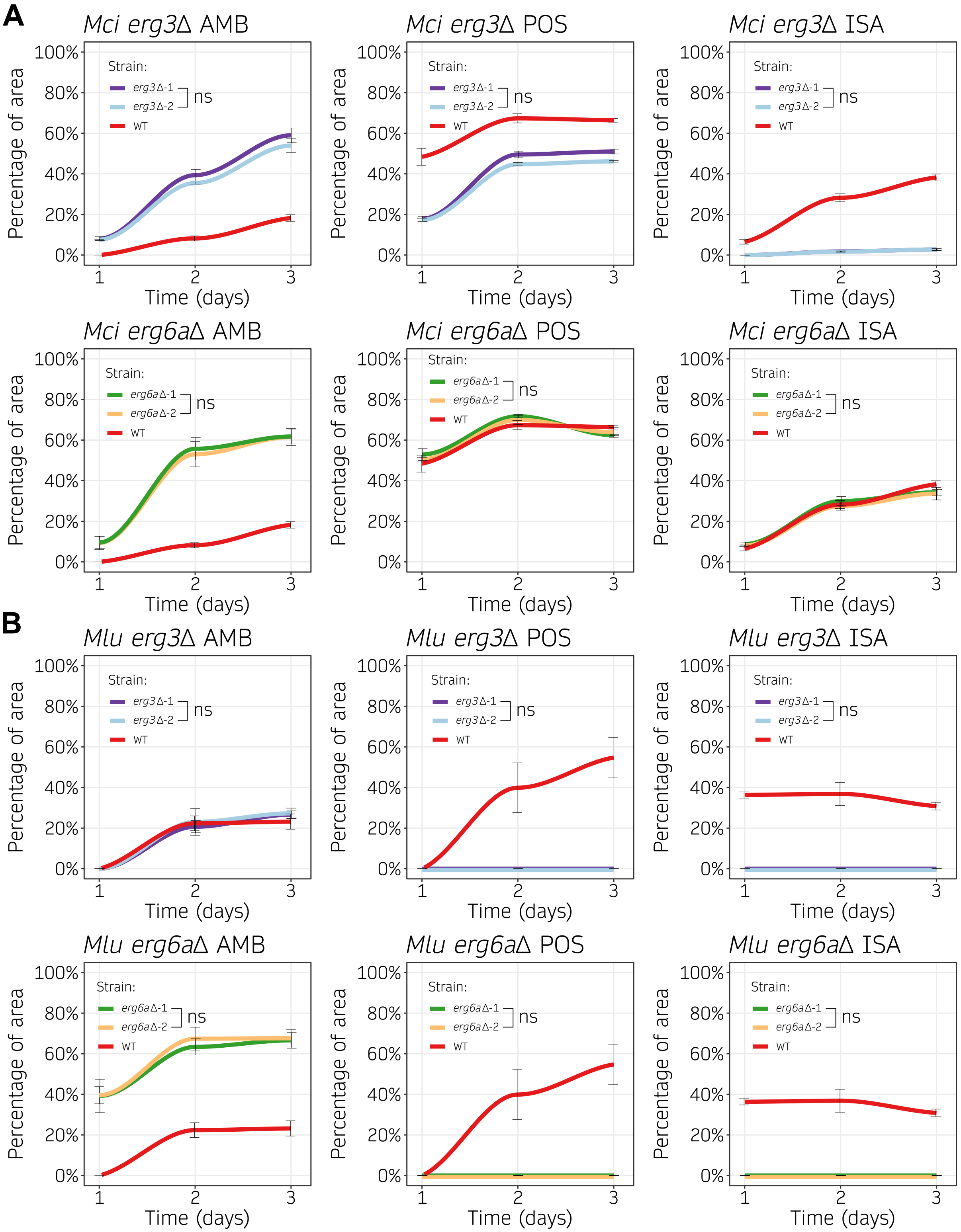

Figure S8. Growth rate during antifungal exposure in *Mucor* species *erg3*Δ and *erg6a*Δ independently generated mutants. Growth across time (24-hour intervals) from independently generated *erg3*Δ and *erg6a*Δ mutants (Δ-1 and Δ-2) in medium containing amphotericin B (AMB, 8 mg/L), posaconazole (POS, 0.2 mg/mL), or isavuconazole (ISA, 8 mg/mL) was determined as the percentage of growth area from the same strain cultured in control medium without drug. Each species was assayed at its optimal temperature, 30 °C and 26 °C for *M. circinelloides* (*Mci*) and *M. lusitanicus* (*Mlu*), respectively. Individual values were collected either in six biological replicates for the wild type or in three biological replicates from each of the two independently generated mutants to plot a color-coded smoothed curve and SD values (black lines). Independently generated mutants exhibiting the same deletion did not show significant differences (ns, One-way ANOVA and Tukey HSD test, p-value $\leq$ 0.05).

Figure S9

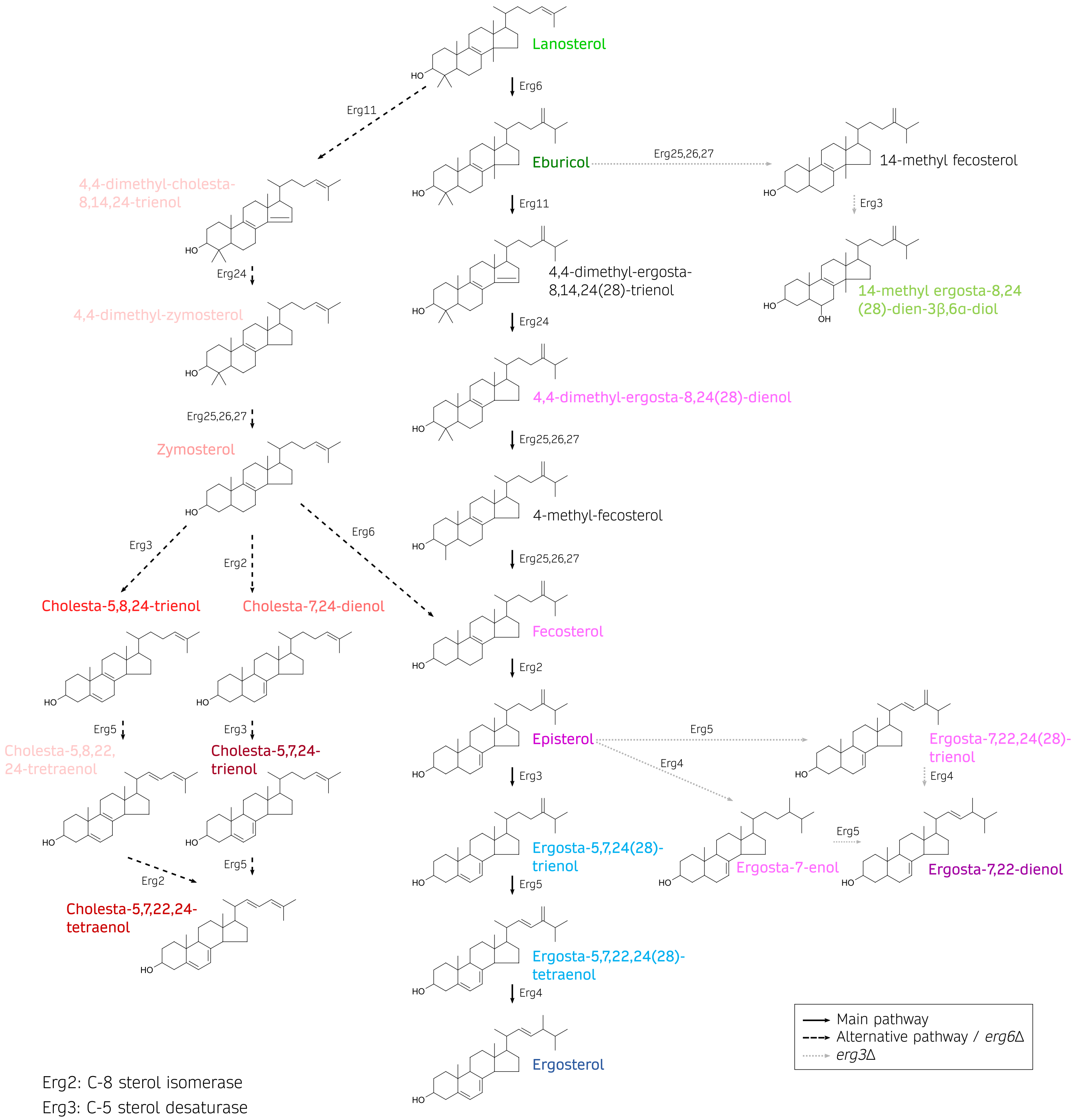

Erg2: C-8 sterol isomerase
Erg3: C-5 sterol desaturase
Erg4: C-24 sterol reductase
Erg5: C-22 sterol desaturase
Erg6: C-24 sterol methyltransferase
Erg11: C-14 sterol demethylase
Erg24: C-14 sterol reductase
Erg25: C-4 sterol methyloxidase
Erg26: C-3 sterol dehydrogenase
Erg27: C-3 ketosteroid retoreductase

Figure S9. Model of the ergosterol biosynthetic pathway in Mucorales. Pathway schematics showing the compounds (line-angle formula and systematic name) and enzymes (enzymatic activity at bottom legend) comprising the ergosterol biosynthetic pathway in Mucorales. The preferred pathway is depicted by straight, black arrows. Alternative pathways that are relevant in *erg3* (dashed, black arrows) or *erg6a* deletions (dotted, gray arrows) are also shown. For better illustration, sterol compounds are classified and color-coded as in Figure 5A.

Figure S10

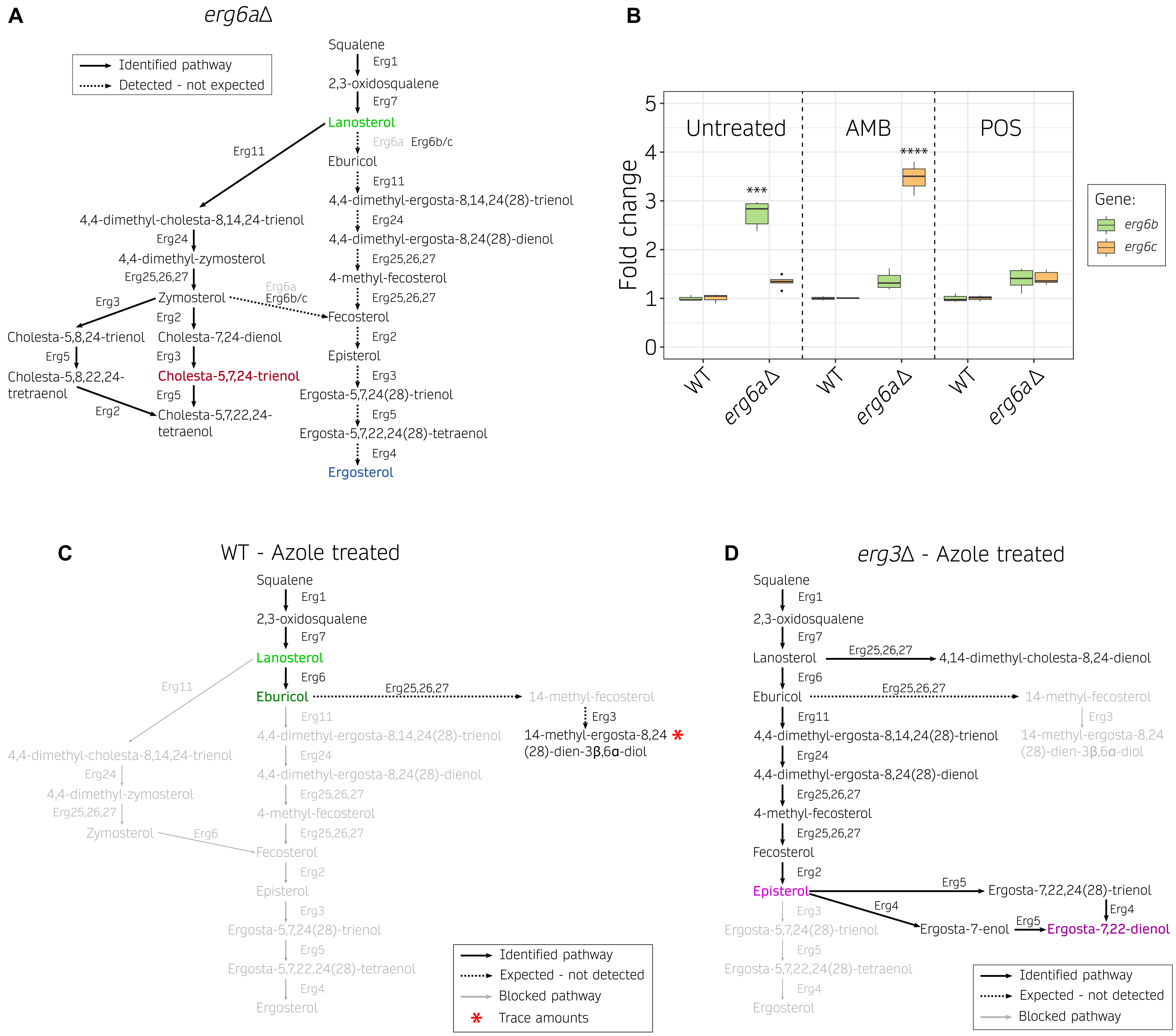

Figure S10. Alternative sterol biosynthetic pathways during azole and amphotericin B treatment in *M. circinelloides*. **(A)** Cholesta-type sterol accumulation in the *erg6a*Δ mutant with amphotericin B treatment. Erg6a loss-of-function is indicated with gray color, and leads to the accumulation of identified cholesta-type sterols defined by straight, black arrows. Unexpected, residual Erg6 activity was detected by the accumulation of C-24 methylated sterols defined by dashed, black arrows. These sterols, including ergosterol, indicate residual Erg6 activity possibly due to additional Erg6 paralogs, Erg6b or Erg6c. **(B)** Box plot of *erg6b* and *erg6c* transcription differences due to *erg6a*Δ mutation compared to the wildtype strain in untreated conditions and upon amphotericin B (AMB) and posaconazole (POS) exposure at each strain half MIC values. Fold-change differential expression levels were quantified by RT-qPCR and normalized to *vma1*, a vacuolar ATPase encoding gene that is constitutively expressed, as an internal control. Minimum, maximum, quartile, and median values correspond to technical triplicates of wildtype, *erg6a*Δ-1, and *erg6a*Δ-2 strains; *erg6a*Δ mutant strain values were pooled together into a total of 6 replicates after no significant differences were observed between them. Significant differences are denoted by asterisks (*** for $P \leq 0.005$ and **** for $P \leq 0.0001$ in a One-way ANOVA and Tukey HSD test).**(C, D)** Accumulation of 14-methylated sterols and other ergosta-type sterols during posaconazole and isavuconazole (azoles) exposure. Identified compounds and reactions are indicated with black arrows, while unidentified compounds and reactions are shown as gray arrows (blocked pathway). Blockades due to azole-mediated Erg11 inhibition in the wildtype strain **(B)** and in *erg3*Δ mutants **(C)** are depicted. Dashed, black arrows indicate the expected accumulation of 14-methyl-fecosterol and the toxic 3,6-diol, but only trace amounts of the toxic 3,6-diol were detected in the wild-type strain during azole treatment. The most abundant sterol compounds in each pathway are color-coded as follows: green (14-methylated sterols), pink (C-5(6)-saturated ergosta-type sterols), red (cholesta-type sterols), and blue (C-5(6)-desaturated ergosta-type sterols).

Figure S11

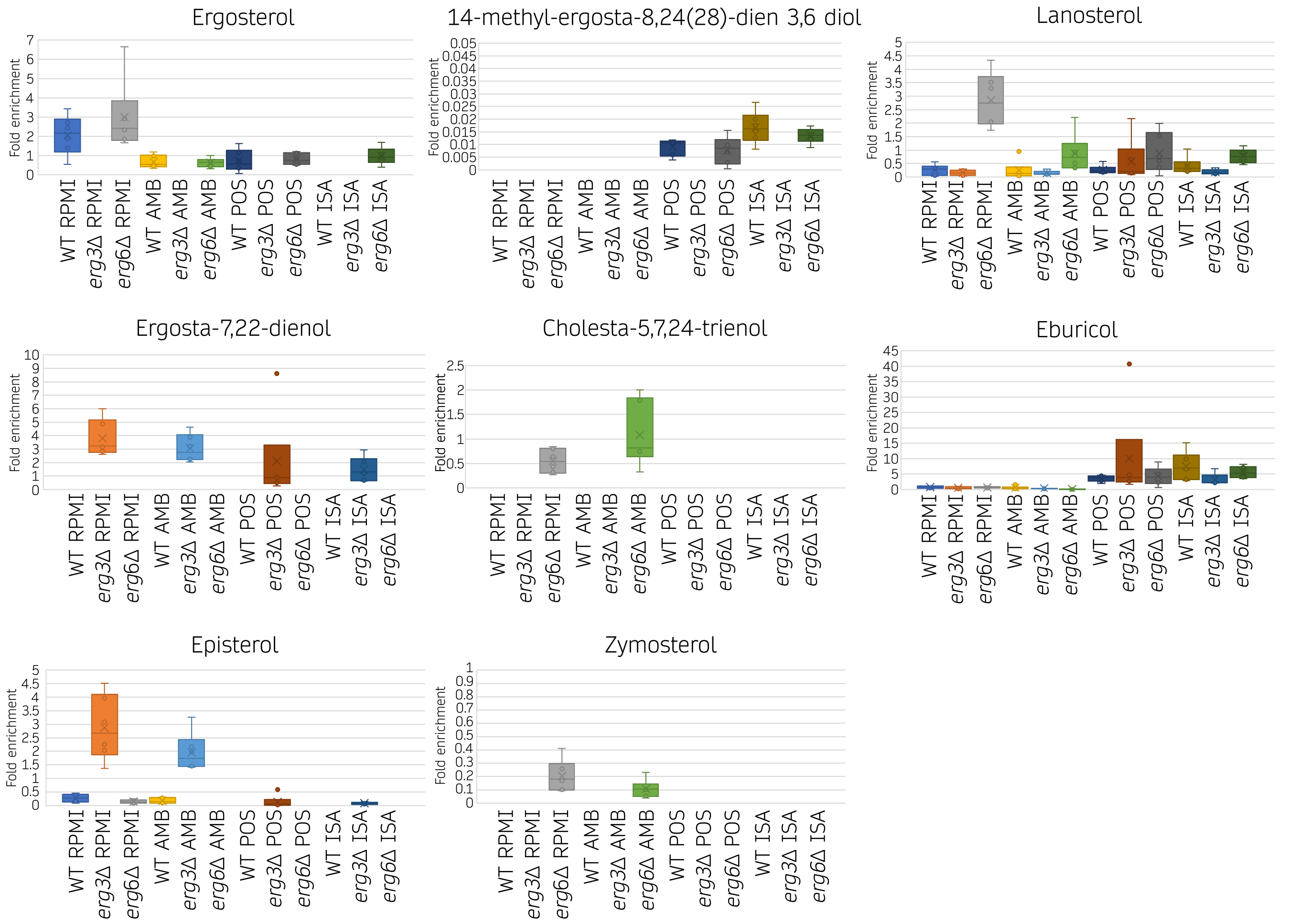

Figure S11. Normalized sterol abundance in *M. circinelloides erg3*Δ and *erg6a*Δ mutants upon amphotericin B and azole treatments. Fold enrichment of different sterols content compared to an internal standard and normalized by the dry weight of each sample. Values were determined from 6 biological replicates, 3 from each pair of independently generated mutant for each deletion, to calculate box plots.
