# Supplementary material for: Alternative ergosterol biosynthetic pathways confer antifungal drug resistance in the human pathogens within the Mucor species complex
Source: mBio. 2024 Jul 9;15(8):e01661-24. doi: 10.1128/mbio.01661-24 (PMC11323496; doi:10.1128/mbio.01661-24)
Supplement: Supplemental Text — Detailed overview of materials and methods. [file mbio.01661-24-s0003.pdf]

Alternative ergosterol biosynthetic pathways confer antifungal drug resistance in the human pathogens within the *Mucor* species complex

María Isabel Navarro-Mendoza[1,4,✉], Carlos Pérez-Arques[1,4,✉], Josie Parker[2], Ziyan Xu[1], Steven Kelly[3], and Joseph Heitman[1,✉]

[1]Department of Molecular Genetics and Microbiology, Duke University School of Medicine, NC 27710, USA
[2]Molecular Biosciences Division, School of Biosciences, Cardiff University, Cardiff, Wales, UK
[3]Institute of Life Science, Swansea University Medical School, Swansea, Wales, UK
[4]Authors contributed equally

Correspondence:

heitm001@duke.edu, maribel.navarro@duke.edu, carlos.parq@duke.edu

Supplementary information

Extended materials and methods

Fungal strains and culturing conditions. *M. lusitanicus* mutant strains generated in this work originate from the *M. lusitanicus* CBS277.49 strain. Its derivative, double auxotrophic (Ura-, Leu-) MU402 strain (75) was used as recipient for genetic transformation procedures, using the selectable marker *pyrG* to complement uracil auxotrophy. The MU636 strain (35) was used as the wildtype, complemented control (*pyrG*⁻::*pyrG*⁺ *leuA*⁻) for phenotypic screening of *M. lusitanicus* mutants. Similarly, *M. circinelloides* mutant strains derive from *M. circinelloides* 1006PhL (39). A uracil auxotroph amenable to genetic transformation, MIN6 (Ura-) was generated in this work. Uridine auxotrophy was complemented by the selectable marker *pyrF*. For phenotypic analysis, the CPA6 strain (*pyrF*⁻::*pyrG*⁺) was used as a control. All the strains generated in this study are listed in Table S3.

The growth rate of *erg3* and *erg6a* mutants was quantified by spot inoculating 500 spores onto rich yeast-peptone-dextrose (YPD) medium and minimal yeast-nitrogen-base (YNB) medium at 26 °C, 30 °C, and 37 °C. Images were taken every 24 hours for 3 days, and analyzed with Fiji (76) to determine the area of growth.

Isolation of uracil auxotrophic strain MIN6. 10^6 spores of the wildtype 1006PhL strain were spread-plated on YPD medium containing 5-FOA (3 g/L) supplemented with uracil (100 mg/L) and uridine (200 mg/L) and screened for resistant isolates after 3 days. Spores from resistant colonies were inoculated onto the same medium for a complete vegetative cycle, i.e. germination, colony formation, and asexual sporangiophore development (4-6 days). Colonies exhibiting stable 5-FOA resistance were inoculated onto YNB media with or without uracil and uridine as aforementioned to determine uracil auxotrophy. Genomic DNA from isolates that exhibited uracil auxotrophy was purified using the MasterPure™ Complete DNA and RNA Purification Kit (Lucigen). The promoter, ORF, and terminator containing sequences from *pyrG* and *pyrF* were PCR-amplified and Sanger-sequenced using primers listed in Table S4.

Protoplast generation and genetic transformation by electroporation. Protoplast generation and transformation by electroporation of *M. lusitanicus* was performed following previously established protocols (48). For *M. circinelloides* transformation, batches of 1.25 x 10^8 MIN6 spores were incubated in 25 mL of yeast-peptone-glucose (YPG) medium at 26 °C for 4 to 4.5 hours, specifically until germlings appeared. Cell walls were digested with a 0.01 M sodium phosphate buffer and 0.5 M sorbitol solution (48) containing 5 mg of lysing enzymes from *Trichoderma harzianum* (L-1412, Sigma-Aldrich) and 0.3 µL (15 U/mg) of chitosanase (C-0794, Sigma-Aldrich) at 30 °C for 1.5 hours, ensuring gentle shaking to avoid protoplast lethality. Protoplasts were washed with cold 0.5 M sorbitol solution, centrifuged at low speed ($\leq$ 1,000 rpm), and resuspended in 800 µL of 0.5 M sorbitol. After that, 200 µl of protoplasts were distributed into electroporation cuvettes, mixed with linear DNA (3-6 µg) and subjected to exponential decay waveform electroporation. Electroporation parameters were set to a field strength of 1,000 V ($E = 5kv \cdot cm^{-1}$), capacitance of 25 µF, and constant resistance of 400 Ω. Following electroporation, protoplasts were recovered in 1 mL of YPG medium, incubated at 26 °C for 1 hour, and spread-plated on minimal medium with casamino acids for *Mucor* (MMC) pH = 3.2 to select prototroph transformants after 2-5 days incubation in dark conditions.

For the deletion of *erg* genes in *M. lusitanicus* we designed a linear DNA construct containing the selectable marker *pyrG* flanked by 1-kb upstream and downstream regions of either the *erg3* or *erg6a* gene. For *M. circinelloides*, the linear DNA construct was similar but contained the selectable marker *pyrF*. In addition, a linear DNA fragment containing the wildtype *pyrF* marker sequence was used to transform MIN6 (*pyrF*-Δ59) and replace the *pyrF* mutated allele. This allowed us to 1) determine transformation efficiencies at a range of voltages and select the optimum to conduct subsequent electroporation experiments, and 2) generate a complemented control named CPA6 (*pyrF*-Δ59::*pyrF*⁺). Gene replacements or deletions through integration by homologous recombination of the DNA cassette as well as homokaryosis were verified by PCR-amplification using primers that generate discriminatory amplicons (AFLP); first, transformants were analyzed after two vegetative passages in selective medium MMC, and subsequently, after every additional passage until homokaryosis was achieved.

Ortholog search and phylogenetic tree inference. Erg3 and Erg6 protein sequences from *Saccharomyces cerevisiae* were used as queries in a PSI-BLAST v2.12.0 search (77) against a proteomic database encompassing 13 mucoralean species known to cause animal or plant disease, the chytrid *Spizellomyces punctatus* as an outgroup, as well as four well-known fungal pathogens that harbored *erg3* and *erg6* single copy genes, *Schizosaccharomyces pombe* and *S. cerevisiae* (see Table S5 for a comprehensive list of species and proteomes) (37–39, 45–47, 78–90). The glomeromycete *Rhizophagus irregularis* Erg6 homologs were also identified to clarify the phylogenetic inference. After that, every match was subjected to a reciprocal BLASTp search against the *S. cerevisiae* proteome, and positive reciprocal hits were retrieved. These putative orthologs (Tables S1 and S2) were aligned using MAFFT v7.475 (91), the resulting alignments were trimmed with TrimAl v1.4.rev15 gappyout method (92), and phylogenetic trees with 1,000 ultrafast bootstraps and SH-aLRT replicates were inferred from these alignment blocks by IQ-TREE v2.2.0.3 (93), detecting the best substitution models automatically.

RNA isolation, sequencing, and data analysis. 2.5 x 10^5 spore/mL YPD cultures were grown for 16 hours at 26 °C and 250 rpm in duplicates. Total RNA was purified with a QIAGEN miRNeasy Mini Kit. rRNA-depleted RNA libraries were prepared using Illumina Stranded Total RNA Prep with Ribo-Zero Gold rRNA Removal Kit and *M. circinelloides* rDNA specific

probes, and cDNA sequenced in a NovaSeq 6000 sequencing system to obtain 150-bp paired-end reads. In addition to these, *M. lusitanicus* similar and publicly available rRNA-depleted RNA sequencing reads were used for gene expression analyses (57). FASTQ dataset quality was assessed by FASTQC v0.11.9 and reads processed by TrimGalore! V0.6.7 to remove adapters and low-quality reads. Processed reads were aligned to either *M. circinelloides* 1006PhL (https://fungidb.org/fungidb/app/record/dataset/DS_8b08c1c31d) or *M. lusitanicus* MU402 genome (https://mycocosm.jgi.doe.gov/Muccir1_3/Muccir1_3.info.html) employing STAR v.2.7.10a (94). Alignments were sorted and classified into forward and reverse stranded by Samtools sort, view, and merge according to their flag values as follows: forward alignments included first-pair alignments from the reverse strand (-f 64 and 16) and second-pair alignments excluding those from the reverse strand (-f 128 and -F 16), and reverse alignments included second-pair alignments from the reverse strand (-f 128 and 16) and first-pair alignments excluding those from the reverse strand (-f 64 and -F 16). Then, coverage files were generated using bamCompare from Deeptools2 v3.5.1 (95) to merge duplicates into a single bigWig file.

Synteny analysis. Pairwise synteny among closely related mucoralean species was assessed at the amino acid level using the JCVI toolkit MCScan pipeline (96), defining synteny range to a maximum of 30 kilobases and a minimum of 2 anchors per cluster. Genomic plots were generated by Deeptools2 pyGenomeTracks v3.7 (97) and protein synteny was manually annotated as pink Bezier curves between species. Genes were plotted as single blocks, i.e. ignoring intron-exon information to facilitate rendering and visualization.

Virulence and fungal burden assays. 4-week old BALB/c mice (Charles River) weighing approximately 20 to 25 g were used as a host model for virulence assays. Mice were immunosuppressed with cyclophosphamide via intraperitoneal injection (200 mg/kg of body weight), 2 days prior to infection and every 5 days thereafter. Groups of 10 mice were intravenously challenged via retro-orbital injection with 1 x 10^6 spores from each of the mutant and control strains. Survival and disease symptomatology of each group of mice were monitored every 12 hours, and animals meeting end-point criteria were euthanized by CO_2 inhalation and a secondary method.

Fungal burden was quantified in three infected mice meeting end-point criteria per group. Animals were dissected to extract five organs (brain, lung, spleen, kidney, and liver), which were homogenized. Organ homogenates were plated on YPD media supplemented with 1 μg/mL of FK506 to induce yeast growth and were incubated for 4 days. Colony forming units (CFU) were quantified and normalized per volume plated and organ weight.

Antifungal drug susceptibility testing. Antifungal drug susceptibility profiles were determined by broth microdilution using the CLSI and EUCAST standard methodology for molds. Briefly, minimal inhibitory concentrations required to inhibit visible growth by eye (MIC) were evaluated for amphotericin B, posaconazole, and isavuconazole. 10^5 spores/mL were incubated in Roswell Park Memorial Institute (RPMI) 1640 medium with each drug at 35 °C for 24 and 48 hours. Results were read visually and wells evaluated for visible growth or lack therein. Additionally, 500 spores were spot-inoculated onto YPD solid medium containing drugs at defined concentrations: 8 mg/L of liposomal amphotericin B (Ambisome, Gilead Sciences), 0.2 mg/L of posaconazole (Noxafil, Merck), and 8 mg/L of isavuconazole (Cresemba, Astellas Pharma). Images of growth were taken at 24-hour intervals, and analyzed with Fiji to measure the area of growth. The area of inhibited growth was determined as the ratio of growth area in treated compared to untreated plates.

Ergosterol profile quantification. Samples were obtained by growing 10^4 spores of the mutant and wildtype strain in RPMI media (untreated) and RPMI supplemented with half the MIC for each corresponding strain for amphotericin B, posaconazole and isavuconazole drugs. Cultures were grown at 35 °C for 48 hours and 60 rpm. Non-saponifiable lipids were extracted from lyophilized mycelia as previously described (12), with cholesterol added as an internal standard. Sterols were derivatized using 0.1 mL *N,O*-Bis(trimethylsilyl)trifluoroacetamide and trimethylsilyl chloride [BSTFA and TMCS, (99:1)] and 0.3 mL anhydrous pyridine and heating at 80 °C for 2 hours (98). TMS-derivatized sterols were analyzed using gas chromatography–mass spectrometry (GS/MS) (Thermo 1300 GC coupled to a Thermo ISQ mass spectrometer, Thermo Scientific) and identified with reference to relative retention times, mass ions and fragmentation spectra. GC/MS data files were analyzed using Xcalibur software (Thermo Scientific). Sterol composition was calculated from peak areas, as a mean of 3 replicates per independently generated mutant (6 replicates per gene deletion) or 6 replicates for the wildtype strain 1006PhL. The relative quantity of sterols present was determined from the peak areas of the sterol and the internal standard and divided by the dry weight of the sample.

RT-qPCR analysis. Cultures were obtained using the same conditions as for ergosterol profiling. Total RNA was isolated as previously described, and cDNA synthesized using Maxima™ H Minus cDNA Synthesis Kit (Thermo Scientific). qPCRs were prepared with SYBR green PCR master mix (Applied Biosystems) using primers that specifically amplified *erg6b* and *erg6c* paralogs, and the vma1 gene served as the endogenous control (Table S4), in triplicate, and performed in a QuantStudio™ 3 real-time PCR system.

Data availability. Raw rRNA-depleted RNA-sequencing datasets obtained from the *M. circinelloides* 1006PhL strain will be accessible upon publication under the following NCBI's Sequence Read Archive (SRA) project accession number: PRJNA1046487. RNA-seq datasets derived from the *M. lusitanicus* MU402 strain were similarly generated for a prior study (57) and are publicly available, assigned to the NCBI's SRA project accession number: PRJNA903107.
