# Supplementary material for: Alternative ergosterol biosynthetic pathways confer antifungal drug resistance in the human pathogens within the Mucor species complex
Source: mBio. 2024 Jul 9;15(8):e01661-24. doi: 10.1128/mbio.01661-24 (PMC11323496; doi:10.1128/mbio.01661-24)
Supplement: Supplemental Tables — Tables S1-S5. [file mbio.01661-24-s0004.pdf]

Table S1

Name	Species	JGI Protein ID	FungiDB Gene ID	EnsemblFungi Gene ID
Erg3	*Candida albicans* SC5314	59490	C1_04770C_A	C1_04770C_A
Erg3	*Choanephora cucurbitarum* NRRL2744	516289		
Erg3	*Cunninghamella echinulata* NRRL1382	185651		
Erg3	*Cryptococcus neoformans* H99	446	CNAG_00519	CNAG_00519
Erg3a	*Lichtheimia corymbifera* JMRC:FSU:9682	10330	LCOR_09389.1	LCOR_09389.1
Erg3b	*Lichtheimia corymbifera* JMRC:FSU:9682	4998	LCOR_04579.1	LCOR_04579.1
Erg3a	*Lichtheimia ramosa* JMRC:FSU:6197	7192		LRAMOSA02887
Erg3b	*Lichtheimia ramosa* JMRC:FSU:6197	11099		LRAMOSA06288
Erg3	*Malassezia pachydermatis* CBS1879	2056	Malapachy_0912	Malapachy_0912
Erg3	*Mucor circinelloides* 1006PhL	6367	HMPREF1544_06438	HMPREF1544_06438
Erg3	*Mucor lusitanicus* MU402	1381623		
Erg3	*Mucor lusitanicus* CBS277.49 v2.0	156917	QYA_156917	MUCCIDRAFT_156917
Erg3	*Mucor racemosus* UBOCC-A-109155	3973		
Erg3	*Phycomyces blakesleeanus* NRRL1555	178901	PHYBL_178901	
Erg3a	*Rhizopus azygosporus* CBS357.93			CU097_003763
Erg3b	*Rhizopus azygosporus* CBS357.93			CU097_010180
Erg3a	*Rhizopus delemar* 99-880	4689	RO3G_07367	RO3G_07367
Erg3b	*Rhizopus delemar* 99-880	7668	RO3G_13407	RO3G_13407
Erg3	*Rhizopus microsporus* var. *microsporus* ATCC52813	213209		RHIMIDRAFT_213209
Erg3	*Saccharomyces cerevisiae* S288C	4075	YLR056W	YLR056W
Erg31	*Schizosaccharomyces pombe* 972h-	372	SPAC1687.16c	SPAC1687.16c
Erg32	*Schizosaccharomyces pombe* 972h-	2860	SPBC27B12.03c	SPBC27B12.03c
Erg3	*Spizellomyces punctatus* DAOM BR117	5830	SPPG_02338	SPPG_02338
Erg3	*Syncephalastrum racemosum* NRRL2496	499858		
Erg3a	*Sporodiniella umbellata* MES 1446	444990		
Erg3b	*Sporodiniella umbellata* MES 1446	496186		
Erg3	*Ustilago maydis* 521	10884	UMAG_03593	UMAG_03593

Table S1. Erg3 amino acid sequences obtained from the genomes of listed fungal species. Identification numbers from different databases [Joint Genome Institute MycoCosm (JGI, https://mycocosm.jgi.doe.gov), Fungi Database (FungiDB, https://fungidb.org) and Ensembl Fungi (https://fungi.ensembl.org] are indicated to facilitate accessibility and reproducibility.

Table S2

Name	Species	JGI Protein ID	FungiDB Gene ID	EnsemblFungi Gene ID
Erg6	*Candida albicans* SC5314	57503	C3_02150C_A	C3_02150C_A
Erg6a	*Choanephora cucurbitarum* NRRL2744	208609		
Erg6b	*Choanephora cucurbitarum* NRRL2744	449520		
Erg6a	*Cunninghamella echinulata* NRRL1382	280554		
Erg6	*Cryptococcus neoformans* H99	3420	CNAG_03819	CNAG_03819
Erg6a	*Lichtheimia corymbifera* JMRC:FSU:9682	1250	LCOR_01192.1	LCOR_01192.1
Erg6b	*Lichtheimia corymbifera* JMRC:FSU:9682	3633	LCOR_03386.1	LCOR_03386.1
Erg6d	*Lichtheimia corymbifera* JMRC:FSU:9682	3987	LCOR_03695.1	LCOR_03695.1
Erg6a	*Lichtheimia ramosa* JMRC:FSU:6197	1343		LRAMOSA06893
Erg6b	*Lichtheimia ramosa* JMRC:FSU:6197	536		LRAMOSA00533
Erg6d	*Lichtheimia ramosa* JMRC:FSU:6197	2430		LRAMOSA07977
Erg6	*Malassezia pachydermatis* CBS1879	2313	Malapachy_1146	Malapachy_1146
Erg6a	*Mucor circinelloides* 1006PhL	10718	HMPREF1544_10842	HMPREF1544_10842
Erg6b	*Mucor circinelloides* 1006PhL	8587	HMPREF1544_08689	HMPREF1544_08689
Erg6c	*Mucor circinelloides* 1006PhL	9624	HMPREF1544_09736	HMPREF1544_09736
Erg6a	*Mucor lusitanicus* MU402	1319648		
Erg6b	*Mucor lusitanicus* MU402	1325190		
Erg6c	*Mucor lusitanicus* MU402	1448211		
Erg6a	*Mucor lusitanicus* CBS277.49 v2.0	155859	QYA_155859	MUCCIDRAFT_155859
Erg6b	*Mucor lusitanicus* CBS277.49 v2.0	151310	QYA_151310	MUCCIDRAFT_151310
Erg6c	*Mucor lusitanicus* CBS277.49 v2.0	74496	QYA_74496	MUCCIDRAFT_74496
Erg6a	*Mucor racemosus* UBOCC-A-109155	9296		
Erg6b	*Mucor racemosus* UBOCC-A-109155	5625		
Erg6c	*Mucor racemosus* UBOCC-A-109155	11127		
Erg6a	*Phycomyces blakesleeanus* NRRL1555	128748	PHYBL_128748	
Erg6b	*Phycomyces blakesleeanus* NRRL1555	114967	PHYBL_114967	
Erg6a	*Rhizopus azygosporus* CBS357.93			CU097_001869
Erg6a	*Rhizopus delemar* 99-880	9579	RO3G_15767	RO3G_15767
Erg6e	*Rhizopus delemar* 99-880	1008	RO3G_16049	RO3G_16049
Erg6a	*Rhizophagus irregularis* DAOM 197198	1517096		
Erg6b	*Rhizophagus irregularis* DAOM 197198	1668347		
Erg6a	*Rhizopus microsporus* var. *microsporus* ATCC52813	203690		RHIMIDRAFT_203690
Erg6	*Saccharomyces cerevisiae* S288C	4664	YML008C	YML008C
Erg6	*Schizosaccharomyces pombe* 972h-	3100	SPBC16E9.05	SPBC16E9.05
Erg6	*Spizellomyces punctatus* DAOM BR117	8734	SPPG_05059	SPPG_05059
Erg6a	*Syncephalastrum racemosum* NRRL2496	499603		
Erg6b	*Syncephalastrum racemosum* NRRL2496	466111		
Erg6a	*Sporodiniella umbellata* MES 1446	459696		
Erg6e	*Sporodiniella umbellata* MES 1446	469068		
Erg6	*Ustilago maydis* 521	10455	UMAG_03182	UMAG_03182

Table S2. Erg6 amino acid sequences obtained from the genomes of listed fungal species. Identification numbers from different databases [Joint Genome Institute MycoCosm (JGI, https://mycocosm.jgi.doe.gov), Fungi Database (FungiDB, https://fungidb.org) and Ensembl Fungi (https://fungi.ensembl.org] are indicated to facilitate accessibility and reproducibility.

Table S3

Strain	Species	Genotype	Reference
MU402	*M. lusitanicus*	*pyrG*⁻ *leuA*⁻	(75)
MU636	*M. lusitanicus*	*pyrG*⁻::*pyrG*⁺ *leuA*⁻	(35)
MIN11	*M. lusitanicus*	*erg3*::*pyrG leuA-*	This study
MIN12	*M. lusitanicus*	*erg3*::*pyrG leuA-*	This study
MIN13	*M. lusitanicus*	*erg6a*::*pyrG leuA-*	This study
MIN14	*M. lusitanicus*	*erg6a*::*pyrG leuA-*	This study
1006PhL	*M. circinelloides*	wildtype	(39)
MIN6	*M. circinelloides*	*pyrF*-59Δ	This study
CPA6	*M. circinelloides*	*pyrF*-59Δ::*pyrF*⁺	This study
MIN15	*M. circinelloides*	*erg3*::*pyrF*	This study
MIN16	*M. circinelloides*	*erg3*::*pyrF*	This study
MIN17	*M. circinelloides*	*erg6a*::*pyrF*	This study
MIN18	*M. circinelloides*	*erg6a*::*pyrF*	This study

Table S3. Strains used in this study, showing from which species they derive, genotype, and references.

Table S4

Name	Species	Sequence (5'⟶3')	Description
JOHE51488	*Mlu*	TGCCTCAGCATTGGTACTTG	*pyrG* PCR
JOHE51489	*Mlu*	GTACACTGGCCATGCTATCG	*pyrG* PCR
JOHE51528	*Mlu*	CGATAGCATGGCCAGTGTACTAGAGTTTACGATGCAGGCCAGT	*erg3* deletion, overlap with *pyrG*
JOHE51539	*Mlu*	CAAGTACCAATGCTGAGGCAATTTCTGTGTGTCAGTCCGCAC	*erg3* deletion, overlap with *pyrG*
JOHE51529	*Mlu*	ATTAGAACAGAAGGGCAGTCGG	*erg3* deletion
JOHE51538	*Mlu*	CGATCATGCTTACGGTGGTTGAGT	*erg3* deletion
JOHE51540	*Mlu*	CGATAGCATGGCCAGTGTACGTGTGCCAAGTGTAGATGTTGTG	*erg6a* deletion, overlap with *pyrG*
JOHE51552	*Mlu*	CAAGTACCAATGCTGAGGCACATCTGTCTCAATATCCGTCGTC	*erg6a* deletion, overlap with *pyrG*
JOHE51541	*Mlu*	ATTTGCCCGCTGTAGATGATAC	*erg6a* deletion
JOHE51551	*Mlu*	AGTGTAGCAAAAGTTGCCCTTG	*erg6a* deletion
JOHE51511	*Mlu*	ACCTTGAGCACACAAACAAAGG	*pyrG,* 5' junction PCR
JOHE51510	*Mlu*	CCTTTGTTTGTGTGCTCAAGGT	*pyrG,* 3' junction PCR
JOHE51533	*Mlu*	ACAGGAAGTGAGTACAACGGACA	*erg3*, 5' junction PCR
JOHE51534	*Mlu*	CATCGCCTCATACTACTCAAAGC	*erg3*, 3' junction PCR
JOHE51531	*Mlu*	TGTGGTATCTACTGGTTCCATCG	*erg3,* WT allele PCR
JOHE51536	*Mlu*	ATGTTGTGTGTCGAGTAGCACC	*erg3,* WT allele PCR
JOHE51545	*Mlu*	GTCTATGTGGCGCTCAATTCTAC	*erg6a*, 5' junction PCR
JOHE51546	*Mlu*	GCGTCCTCTTCCATTCCTACTAC	*erg6a*, 3' junction PCR
JOHE51543	*Mlu*	GCTGGTGATATTTACGAGTCTGC	*erg6a,* WT allele PCR
JOHE51549	*Mlu*	GGAGAGATTTGATGGTGGTGTT	*erg6a,* WT allele PCR
JOHE51498	*Mci*	AGAATGCCAGACCTGAATTTTTGG	*pyrF* PCR
JOHE51499	*Mci*	AATAGTAATACCCTCTGCCAACGG	*pyrF* PCR
JOHE51623	*Mci*	CCGTTGGCAGAGGGTATTACTATTGATATGGTAGCGAACCCGATG	*erg3* deletion, overlap with *pyrF*
JOHE51628	*Mci*	CCAAAAATTCAGGTCTGGCATTCTAATTTCTGTGTGTCAGTCCGCAC	*erg3* deletion, overlap with *pyrF*
JOHE51624	*Mci*	GGATGAGTCTTCAGGTGCAGTAA	*erg3* deletion
JOHE51627	*Mci*	AATCCGAGAATGATGTGTGAATACC	*erg3* deletion
JOHE51630	*Mci*	CCGTTGGCAGAGGGTATTACTATTCAACATCAAATCTCTCCCGTTC	*erg6a* deletion, overlap with *pyrF*
JOHE51634	*Mci*	CCAAAAATTCAGGTCTGGCATTCTCTCAATATCCGTCTTCCCCTTAC	*erg6a* deletion, overlap with *pyrF*
JOHE51629	*Mci*	AAGATTGGAGCACCTCAAGAGAC	*erg6a* deletion
JOHE51633	*Mci*	AGTCGTGCAGCAGGTAGTTTTAG	*erg6a* deletion
JOHE51503	*Mci*	GAGCAGCAGCATAGAAAGTACCA	*pyrF,* 5' junction PCR
JOHE51504	*Mci*	TATCAAGAAGGACTAGGCTTGCC	*pyrF,* 3' junction PCR
JOHE51625	*Mci*	TGTCACCTACACGAATCTTACCC	*erg3*, 5' junction PCR
JOHE51626	*Mci*	AGAGAATATGGCGCTTCAGAGAC	*erg3*, 3' junction PCR
JOHE51625	*Mci*	TGTCACCTACACGAATCTTACCC	*erg3,* WT allele PCR
JOHE51637	*Mci*	TGTTCATCGGTGGGTTGACG	*erg3,* WT allele PCR
JOHE51631	*Mci*	CTGATTTCACAGTACGCCTGAGT	*erg6a*, 5' junction PCR
JOHE51632	*Mci*	GGTAAAGAATATGGGCACAGGTC	*erg6a*, 3' junction PCR
JOHE51631	*Mci*	CTGATTTCACAGTACGCCTGAGT	*erg6a,* WT allele PCR
JOHE51641	*Mci*	GCATCACCAACATTGGCAAG	*erg6a,* WT allele PCR
JOHE51712	*Mci*	GCAAGGCGATATGGGTGTTG	*erg6b,* qPCR
JOHE51713	*Mci*	GACTGAAGAGCGAAGAAACCC	*erg6b,* qPCR
JOHE51714	*Mci*	ACTCAAGCTGGTCGGTATGC	*erg6c,* qPCR
JOHE51715	*Mci*	ACAACAGAATCGCCTGCAAC	*erg6c,* qPCR
JOHE51665	*Mci*	AGGGTCAATACACTCTTGAAG	*vma1,* qPCR
JOHE51667	*Mci*	GGAGAACGAACAGGCCAGAG	*vma1,* qPCR

Table S4. Species-specific primers [*M. lusitanicus* (*Mlu*) or *M. circinelloides* (*Mci*)] used in this study, showing systematic name, nucleotide sequence, and a brief description explaining its use.

Table S5

Species	Database	Reference
Candida albicans SC5314	JGI	(78)
Choanephora cucurbitarum NRRL2744 v1.0	JGI	(79)
Cryptococcus neoformans var. *grubii* H99	JGI	(80)
Cunninghamella echinulata NRRL1382 v1.0	JGI	(79)
Lichtheimia corymbifera JMRC:FSU:9682	JGI	(45)
Lichtheimia ramosa JMRC:FSU:6197	JGI	(81)
Malassezia pachydermatis CBS1879	JGI	(82)
Mucor circinelloides 1006PhL	JGI	(39)
Mucor lusitanicus MU402 v1.0	JGI	(38)
Mucor racemosus UBOCC-A-109155	JGI	(83)
Phycomyces blakesleeanus NRRL1555 v2.0	JGI	(37)
Rhizophagus irregularis DAOM 197198 v2.0	JGI	(84)
Rhizopus azygosporus CBS357.93	EnsemblFungi	(47)
Rhizopus delemar 99-880	JGI	(46)
Rhizopus microsporus var. *microsporus* ATCC52813 v1.0	JGI	(85)
Saccharomyces cerevisiae S288C	JGI	(86)
Schizosaccharomyces pombe 972h-	JGI	(87)
Spizellomyces punctatus DAOM BR117	JGI	(88)
Sporodiniella umbellata MES 1446 v1.0	JGI	(79)
Syncephalastrum racemosum NRRL2496 v1.0	JGI	(89)
Ustilago maydis 521	JGI	(90)

Table S5. Proteomes from these species were obtained from publicly available repositories either at the Joint Genome Institute MycoCosm (JGI, https://mycocosm.jgi.doe.gov) or, if unavailable, at the EnsemblFungi (https://fungi.ensembl.org) databases.
